# SrMo_0.9_O_3−δ_ Perovskite with Segregated Ru Nanoparticles Performing as Anode in Solid Oxide Fuel Cells

**DOI:** 10.1021/acsami.3c19099

**Published:** 2024-04-02

**Authors:** Vanessa Cascos, Mónica Chivite Lacaba, Neven Biskup, María Teresa Fernández-Díaz, José Antonio Alonso

**Affiliations:** † Departamento de Química Inorgánica, Universidad Complutense de Madrid, Madrid E-28040, Spain; ‡ Instituto de Ciencia de Materiales de Madrid, C.S.I.C., Cantoblanco, Madrid E-28049, Spain; § Departamento de Física de Materiales & Instituto Pluridisciplinar, Universidad Complutense de Madrid, Madrid 28040, Spain; ∥ Institut Laue Langevin, BP 156X, Grenoble F-38042, France

**Keywords:** SOFC, perovskite material, Ru exsolution, MIEC anode, neutron diffraction, SrMoO_3_

## Abstract

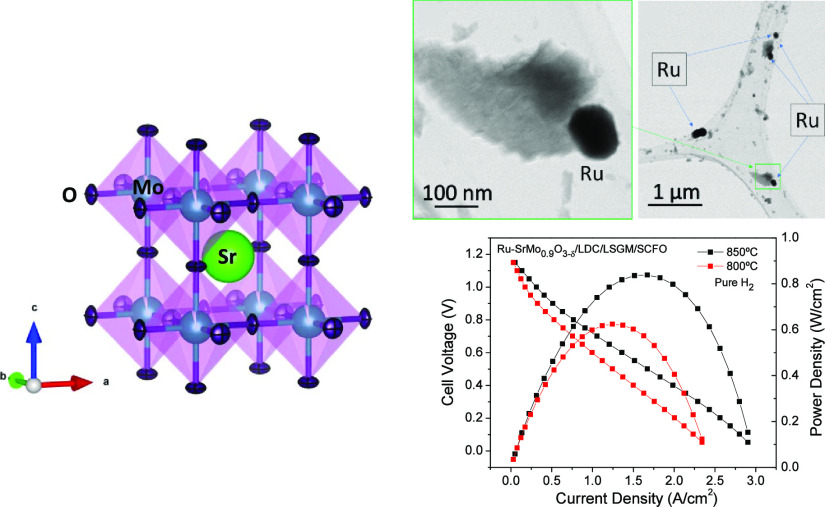

A new anode material, Ru-SrMo_0.9_O_3−δ_, with a perovskite structure and segregated metallic Ru, has been tested in an intermediate-temperature solid oxide fuel cell (IT-SOFC) in an electrolyte-supported configuration giving substantial power densities as high as 840 mW/cm^2^ at 850 °C using pure H_2_ as fuel. This material has been prepared by the citrate method and structurally and microstructurally characterized at room temperature by different techniques such as X-ray diffraction (XRD), neutron powder diffraction (NPD), scanning electron microscopy (SEM), and scanning transmission electron microscopy (STEM). NPD was very useful to determine oxygen positions and vacancies, unveiling a cubic and oxygen-deficient perovskite SrMo_0.9_O_3−δ_ oxide with a *Pm-*3*m* space group and potential ionic mobility. On the other hand, SEM and STEM studies have allowed to identify metallic segregated Ru nanoparticles providing the material with an excellent catalytic activity. Other properties such as the thermal expansion coefficient (TEC) and chemical compatibility with other cell components or electrical conductivity have also been studied to understand the excellent performance of this material as anode in IT-SOFC and correlate it with the crystallographic structure.

## 1 Introduction

A solid oxide fuel cell (SOFC) is an electrochemical device that uses a spontaneous thermodynamic reaction (Δ*G*^0^ < 0) to convert the chemical energy contained in a fuel, such as hydrogen or natural gas, into electrical energy and heat with high efficiency and low environmental impact. These devices are characterized by working at high temperatures (around 1000 °C), and all the constituents are metal oxides.^1,2^ This high temperature helps the diffusion of the oxide ions across the solid electrolyte once the oxygen reduction reaction (ORR) is undergone in the cathode material (also a metal oxide);^3^ as a drawback, the high temperatures contribute to the component degradation inside the cell, thus requiring costly materials, like typically interconnectors, raising the price of the cell. For these reasons, lowering the working temperature of the cell to a range between 750 and 850 °C without its performance detriment is a prime target, in the so-called intermediate-temperature SOFC (IT-SOFC).

The basic functional elements in an SOFC are the electrolyte, electrodes (cathode and anode) and interconnectors. In the case of anodes, where the fuel is electrochemically oxidized, the required features are high electronic conductivity, stability in reducing atmospheres, easiness to incorporate oxygen vacancies to obtain ionic conductivity, good catalytic activity for fuel oxidation, good porosity to transport the fuel gas to the electrolyte, no reaction between the anode and the electrolyte and a thermal expansion coefficient (TEC) similar to the rest of cell components. In particular, the metallic SrMoO_3_ perovskite, reduced from its oxidized scheelite phase SrMoO_4_ in a hydrogen atmosphere, possesses most of these characteristics.^4−11^ Unfortunately, the stoichiometric material lacks ionic conductivity. For this reason, this material has been previously doped with different elements such us Fe^3+^, Cr^3+^, Ga^3+^, Co^2+^, Mg^2+^, and Ni^2+^^12−17^ at the Mo(IV) crystallographic positions, in order to incorporate oxygen vacancies and boost the required mixed ionic and electronic conductivity (MIEC). Therefore, SrMo_1–*x*_M_*x*_O_3−δ_ (M = Fe, Cr, Ga, Co, Mg, Ni; *x* = 0.1 and 0.2) cubic perovskites were designed, synthesized, and tested as anode materials,^12−17^ obtaining extraordinary output powers between 600 to 1050 mW/cm^2^ under a pure H_2_ flow at 850 °C. In the case of Ni, this atom was introduced into the scheelite phase by partially replacing Mo, and afterward, Ni particles were successfully exsolved from the structure to the surface of the oxide once the scheelite was reduced to the perovskite phase. The Ni particles remained in exsolution on the surface, promoting an excellent catalytic activity for the hydrogen oxidation reaction (HOR) of this material; in electrolyte-supported single cells, a substantial output power of 1025 mW/cm^2^ was obtained at 850 °C with pure H_2_ as a fuel.^17^ This result opens the possibility of extrapolating these findings to other catalytically active metals in novel M-SrMo_1–*x*_O_3−δ_ composite materials with exsolved or segregated M metal nanoparticles in the surface of the anode oxide matrix.

In this work, 10% Ru was chosen to prepare a Ru-SrMo_0.9_O_3−δ_ perovskite material where, in this case, metallic Ru particles are segregated from the bulk crystal structure to the surface. The introduction of Ru at the Mo positions stabilizes a cubic crystal structure, simultaneously incorporating oxygen vacancies in the material, with a double aim of improving the oxygen diffusion together with the MIEC properties and the catalytic activity. We present the synthesis and the structural and microstructural characterization by XRD, NPD, SEM, and STEM techniques of this novel perovskite member. Different properties such as dilatometric analysis, chemical compatibility, redox reversibility, electrical conductivity, and porosity complement these results. Finally, electrochemical tests demonstrated its significant performance in IT-SOFCs.

## 2 Experimental Section

### 2.1 Synthesis of the Anode Material

SrMo_0.9_Ru_0.1_O_4−δ_ scheelite oxide was prepared by a variant of the citrate route using a stoichiometric mixture of Sr(NO_3_)_2_, (NH_4_)_6_Mo_7_O_24_·4H_2_O, and RuO_2_ powders. Sr(NO_3_)_2_ and (NH_4_)_6_Mo_7_O_24_·4H_2_O were first weighed and dissolved in a 10% solution of citric acid with some drops of nitric acid under constant heating and stirring. RuO_2_ powder was added later and remained in suspension under stirring until all of the solvent was progressively evaporated and a polymer resin was formed. This resin contains the metal ions (Sr, Mo, and Ru) dispersed at the atomic level and was first dried at 150 °C in an oven for 3 h and decomposed at temperatures up to 600 °C for 12 h, obtaining a SrMo_0.9_Ru_0.1_O_4−δ_ scheelite precursor with a high degree of homogeneity. By reduction of the scheelite precursor, a SrMo_0.9_O_3−δ_ cubic perovskite with exsolved Ru-metal particles was obtained under a forming gas flow, H_2_/N_2_ (5%/95%), in a tubular furnace at 1050 °C for 15 h. This reducing atmosphere drives the reduction of Mo^6+^ to Mo^4+^, yielding this Ru-SrMo_0.9_O_3−δ_ biphasic composite.

### 2.2 Structural Characterization

The reaction products were first characterized by X-ray diffraction (XRD) using a Bruker D8 Advanced diffractometer operating at 40 kV and 30 mA, controlled by DIFFRACplus software in a Bragg–Brentano reflection geometry with Cu Kα radiation (λ = 1.5418 Å). XRD data were collected in a 2θ range from 11 to 64°. Besides, the chemical compatibility of Ru-SrMo_0.9_O_3−δ_ with the La_0.8_Sr_0.2_Ga_0.83_Mg_0.17_O_3−δ_ (LSGM) electrolyte and the La_0.4_Ce_0.6_O_2−δ_ (LDC) buffer layer was also tested with this XRD technique.

Neutron powder diffraction (NPD) data at room temperature (RT) were acquired for Ru-SrMo_0.9_O_3−δ_ perovskite, in order to perform a more detailed crystallographic study, in which the amount of oxygen in the structure could be determined. This NPD study was carried out in the D2B diffractometer at the Institut Laue-Langevin, in Grenoble (France), in the high-resolution configuration with a neutron wavelength λ = 1.549 Å. About 2 g of sample was contained in a vanadium can; the measurement was collected at RT. The collection time for the pattern was 2 h. Diffraction data were analyzed by the Rietveld method,^18^ using the FullProf refinement program.^19^ A pseudo-Voigt function generated the profile shape. The following parameters were refined in the final run of the fit: scale factor, background coefficients, zero-point error, unit-cell parameters, pseudo-Voigt corrected for asymmetry parameters, positional coordinates, isotropic thermal factors for the metals, and anisotropic for oxygen atoms. Occupancy factors for oxygen atoms were also refined from NPD data. The coherent scattering lengths of Sr, Mo, Ru, and O are 7.020, 6.715, 7.03, and 5.803 fm, respectively.

### 2.3 Microstructural Characterization

Scanning electron microscopy (SEM) images were carried out with a field-effect FEI Nova NanoSEM 230 microscope for the microstructural characterization and a table-top Hitachi TM-1000 microscope for the postmortem evaluation of the single cell. Transmission electron microscopy was done in scanning (STEM) mode with a JEOL ARM200F microscope. This enabled us to record both annular bright-field (ABF) images and annular dark-field (ADF) images and detect the spatial distribution of chemical composition. Elemental mapping was done using Oxford Instruments electron-dispersive X-ray spectroscopy (EDS).

### 2.4 Thermal Analysis

Thermal analyses were performed in a Mettler TA3000 system equipped with a TC10 processor unit, starting from the reduced perovskite phase. In this experiment, about 50 mg of the sample was treated at a heating rate of 10 °C min^–1^ in an O_2_ flow. The curves were obtained in a TG50 unit heating from 30 to 900 °C.

### 2.5 Thermal Expansion Coefficients

TECs of the perovskite and scheelite phases were obtained from dilatometry experiments. The analysis was carried out on a Linseis L75HX1000 dilatometer on sintered pellets of the oxidized and reduced compositions. The selected atmosphere for the scheelite phase was air, while the perovskite was measured under H_2_/N_2_ (5%/95%) flow, both between 200 and 850 °C with a heating rate of 10 °C min^–1^. Perovskite pellets of around 7 mm diameter and 1.5 mm thickness were prepared by uniaxial pressing of the powders and subsequently annealed at 1050 °C for 12 h in a H_2_/N_2_ (5%/95%) flow, in order to prevent the oxidation of the material. On the other hand, pellets of the scheelite powders were sintered at 1050 °C for 12 h in air.

### 2.6 DC Conductivity

Electrical conductivity measurement was acquired in the temperature range from 25 to 850 °C under H_2_/N_2_ (5%/95%) flow using the dc four-probe method under a dc current applied between 0.01 and 0.5 A. A bar-shaped pellet (∼2 mm × 3 mm × 9 mm) was prepared by uniaxial pressing using 0.3 g of powder with a Specac Manual Hydraulic Press, subsequently annealed at 1050 °C for 12 h under a H_2_/N_2_ (5%/95%) atmosphere. A Potentiostat-Galvanostat AUTOLAB PGSTAT302 from ECO CHEMIE was used to measure the electrical conductivity of the samples collected every 50 °C.

### 2.7 Single-Cell Performance

Ru-SrMo_0.9_O_3−δ_ was tested as anode material in single cells using 300 μm-LSGM pellets as the electrolyte, SrCo_0.8_Fe_0.2_O_3−δ_ (SCFO) as a reference cathode, and La_0.4_Ce_0.6_O_2−δ_ (LDC) as a buffer layer, whose role is to prevent the interdiffusion of ionic species between the electrolyte and the anode. The LSGM material was obtained from the starting materials (La_2_O_3_, SrCO_3_, Ga_2_O_3_, and MgO), following a ceramic method, with sequential heating treatments in air at 1000 and 1200 °C for 20 h in air, with intermediate grinding. The final LSGM pellets were obtained by pressing LSGM powders and then sintering them at 1450 °C in air for 20 h at a ramping rate of 3 °C min^–1^. After the pellets were ground with rotating SiC wheels, the resulting LSGM pellets had a thickness of around 300 μm. Inks of the anode, buffer layer, and cathode were prepared by ball-milling the powders in ethanol to break the agglomerates. The dried powders were mixed with a binder (V-006 from Heraeus) to prepare the inks. A thin layer of buffer layer ink was painted on one side of LSGM in a 0.5 cm × 0.5 cm area and posteriorly calcined at 1300 °C for 1 h. Afterward, a thin layer of anode ink was painted onto the previous buffer layer and subsequently calcined at 1050 °C for 1 h. Finally, the ink of the SCFO cathode was coated onto the other side of the electrolyte with the same dimensions and fired at 1000 °C for 1 h. A Pt grid, provided with Pt wires, was glued to each of the electrodes using Pt paste and afterward calcined at 850 °C for 1 h, which were used as electrical collectors. The cell was evaluated in a vertical tubular furnace at 800 and 850 °C. The anode was sided toward the pure H_2_ flux while the cathode was open to the air. Data were collected in an AUTOLAB 302N Potentiostat/Galvanostat under a changing voltage of the cell from the open circuit value (OCV) to 0.1 V, with steps of 0.010 V, holding for 10 s at each step. Values of the current density were calculated considering the effective working area of the cell (0.25 cm^2^). Each VI (voltage intensity) scan corresponds to one cycle; the activation of the cell was followed by subsequent cycles until the full power of the single cell was reached. Electrochemical impedance spectra (EIS) data were collected using an AUTOLAB FRA system (PGSTAT30 and FRA2 module) from ECO Chemie B.V. The frequency range was set from 1 MHz to 1 Hz with a signal amplitude of 0.05 V. Measurements were performed on the single cell during power determinations.

## 3 Results and Discussion

### 3.1 Crystallographic Characterization

**Figure 1:**
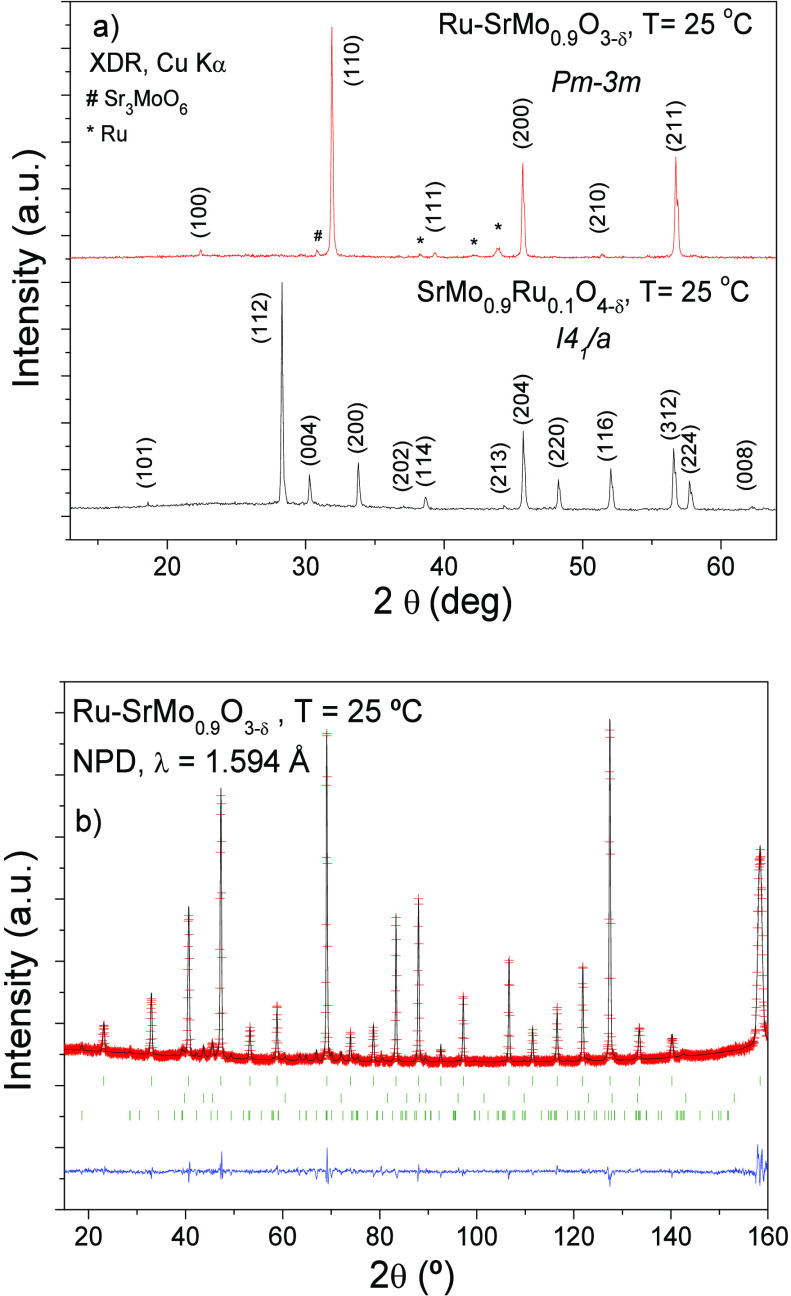
(a) XRD patterns collected with Cu Kα radiation for SrMo_0.9_Ru_0.1_O_4−δ_ (black line) and Ru-SrMo_0.9_O_3−δ_ (red line) samples. The asterisks indicate the presence of metallic Ru. The # symbol indicates a tiny impurity of Sr_3_MoO_6_. (b) Observed (red crosses), calculated (black full line), and difference (blue line) NPD patterns for the Ru-SrMo_0.9_O_3−δ_ sample at 25 °C, refined in the *Pm-3m* space group. A second and third set of Bragg reflections correspond to segregated metallic Ru (s.g. *P*6_3_/*mmc*) and a minor amount of oxidized scheelite phase (s.g. *I4*_*1*_/*a*), respectively.

Both scheelite and perovskite samples obtained by the citrate route were first studied by XRD. This study was used for a primary characterization of the material properties, such as crystal structure and crystallite size. Figure 1a shows the X-ray diffractograms of both oxidized and reduced compounds at 25 °C. First, the oxidized phase with scheelite structure SrMo_0.9_Ru_0.1_O_4−δ_ crystallized in the tetragonal *I*4_1_/*a* space group and was obtained with 10% of Ru introduced into the Mo(VI) position. No impurities were detected in this phase. After the scheelite was reduced in a 5% H_2_/95% N_2_ atmosphere, a cubic perovskite phase Ru-SrMo_0.9_O_3−δ_ with segregated metallic Ru (*s.g*. *P*6_3_/*mmc)* was obtained (Figure 1a). This perovskite crystallized in the cubic *Pm-*3*m* space group and a tiny impurity of Sr_3_MoO_6_ was found (# symbol in Figure 1a).

**Figure 2:**
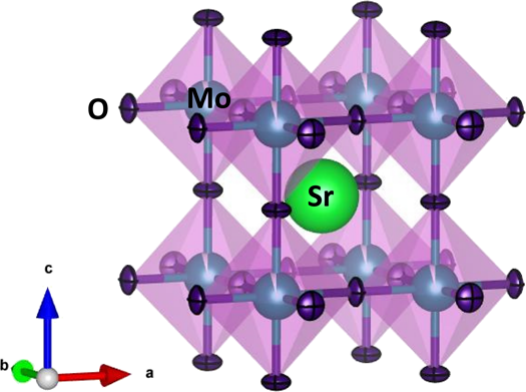
Crystal structure of the SrMo_0.9_O_3−δ_ perovskite matrix showing the oblate-type oxygen ellipsoids at 25 °C. Anisotropic displacement factors for oxygen atoms are represented as ellipsoids with 95% probability.

To analyze the crystal structure of Ru-SrMo_0.9_O_3−δ_ perovskite in more detail, an NPD study at 25 °C in the high-resolution D2B diffractometer (Grenoble, France) was carried out. This technique allows determining occupancies of all the atoms including oxygen, as well as the anisotropic or isotropic displacement factors, which are essential parameters to evaluate a material as an electrode in an SOFC. Ru-SrMo_0.9_O_3−δ_ was successfully refined by the Rietveld method in the cubic *Pm-*3*m* space group at 25 °C, with *Z* = 1. This space group is described with Sr atoms located at 1*b* (1/2, 1/2, 1/2) positions, Mo atoms distributed at random at 1*a* (0, 0, 0) sites, and the O oxygen atoms placed at 3*d* (1/2, 0, 0) positions. The occupancy factors of the molybdenum and oxygen atoms were also refined, and vacancies in both positions were observed at RT. The crystallographic formula after refining these occupancies and considering that all the Ru atoms are segregated to the surface of the perovskite is denoted as Ru-SrMo_0.8720(7)_O_2.736(2)_. This huge amount of oxygen vacancies was expected due to the Mo deficiency, giving rise to a valence state of Mo^3.98+^. The same phenomenology was found in a similar material with exsolved Ni instead of Ru.^17^Figure 1b shows the good agreement between the observed and calculated NPD patterns at 25 °C for Ru-SrMo_0.9_O_3−δ_ oxide, confirming the cubic symmetry with unit-cell parameter *a* = 3.975010 (2) Å. This reduction in the unit-cell parameter with respect to the parent compound without Ru (SrMoO_3_; *a* = 3.9762(3) Å^20^) is probably due to the Mo deficiency in the perovskite matrix together with the oxygen deficiency. The second and third sets of Bragg reflections in Figure 1b correspond to the segregated metallic Ru (s.g. *P*63*/mmc*) and a minor amount of unreduced scheelite phase (s.g. *I*41*/a*), respectively. However, the tiny Sr_3_MoO_6_ impurity found by XRD was not seen with NPD. The anisotropic displacement factors of oxygen atoms were also refined (Figure 2); the Sr and Mo atoms are isotropic by symmetry. Table 1 includes the final crystallographic parameters after Rietveld refinement from NPD data at 25 °C.

**Table 1:** Structural Parameters of the Ru-SrMo_0.9_O_3−δ_ Perovskite after the Rietveld Refinement from NPD Data

**crystal data**	
cubic, *Pm-3m*	NPD, λ = 1.594 Å
*a* = 3.975010 (2) Å	*V* = 62.807 (1) Å^3^

**discrepancy factors**	
*R*_p_ = 3.66%	*R*_wp_ = 4.83%
*R*exp = 1.41%	*R*_Bragg_ = 3.57%
χ^2^ = 11.7	

**fractional atomic coordinates and equivalent isotropic and anisotropic displacement parameters (Å^2^)**					
	***x***	***y***	***z***	*B*_iso_Sr,Mo/*B*_eq_O (Å^2^)	**occ**
**Sr**	0.50000	0.50000	0.50000	0.749(3)	1.00
**Mo**	0.00000	0.00000	0.00000	0.063(2)	0.8720(7)
**O**	0.50000	0.00000	0.00000	0.7833	0.9121(7)

**O anisotropic displacements**	
β_11_^a^	50(7)
β_22_^a^ = β_33_^a^	161(5)

a Anisotropic betas (× 10^4^). β_12_ = β_23_ = β_13_ = 0.

The cubic perovskite structure of Ru-SrMo_0.9_O_3−δ_ after refinement at 25 °C is displayed in Figure 2. It is appreciated a notable anisotropy in the disk-shaped (oblate type) oxygen displacement ellipsoids with 95% probability.

### 3.2 Microstructural Characterization

Highly porous materials outperform their dense counterparts as electrodes due to their enhanced gas permeability, which is particularly beneficial for anode fuel flow. SEM images depict the morphology of the Ru-SrMo_0.9_O_3−δ_ powder, revealing agglomerates comprising small grains that form channels. These structural features facilitate the diffusion and oxidation processes of fuel throughout the anode bulk.

**Figure 3:**
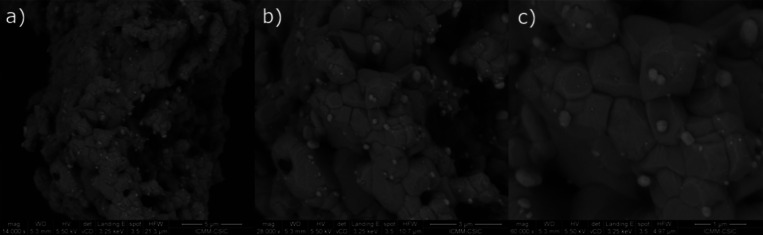
SEM micrographs of Ru-SrMo_0.9_O_3−δ_ powder showing segregated Ru nanoparticles and large pores and channels in the sample with (a) 14,000×, (b) 28,000×, and (c) 60,000× magnifications.

In Figure 3a–c, captured at varying scales, segregated Ru nanoparticles are prominently visible, adhering to the surface of the perovskite matrix. These nanoparticles exhibit a spherical morphology, with sizes characterized by an average distribution diameter of less than 0.1 μm.

Additional pictures are included in the Supporting Information file (Figures S1 and S2 for the oxidized and reduced phases, respectively). In an EDX study (Figures S3–S6), we show that, whereas in the oxidized scheelite-type phase, Ru is homogeneously distributed within the crystalline oxide, in the reduced perovskite phase, Ru is concentrated in the segregated particles, with heterogeneous sizes that emerge from the surface of the perovskite matrix. Moreover, such a perovskite oxide does not have significant Ru amounts. Please notice that, given the limitations of the technique to focalize in a single point, the Ru particles also seem to contain some Sr, Mo, and O, due to the proximity of the oxide matrix.

**Figure 4:**
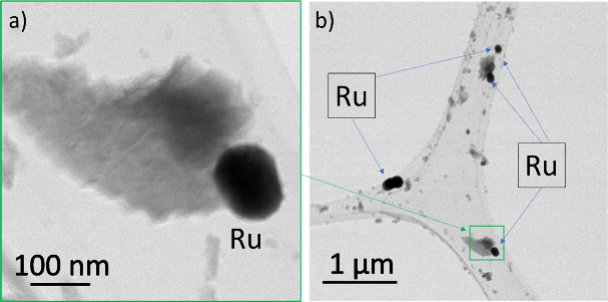
(a, b) STEM micrographs of the Ru–SrMo_0.9_O_3−δ_ sample at different scales, showing segregated Ru nanoparticles. The typical pair of grains in (a) can be seen in the low-magnification image (b). The green square in (b) depicts the grains from Figure 3a.

Figure 4 shows the STEM ABF images obtained for the reduced Ru-SrMo_0.9_O_3−δ_ material for two different magnifications, illustrating segregated Ru nanoparticles together with the perovskite matrix. These nanoparticles present spherical morphology, with different sizes typically smaller than 100 nm. The typical pair of Ru-SrMo_0.9_O_3−δ_ crystallites shown in Figure 4a is also observed at a large scale (Figure 4b). In ABF images (as in conventional TEM images), the grains with higher average atomic numbers are darker. This is how we can differentiate two types of grains: dark grains containing only ruthenium and dimmer grains containing lighter SrMo_0.9_O_3−δ_. To be sure of the chemical composition of our material, an elemental mapping of the two grains shown in Figure 4a has been done.

**Figure 5:**
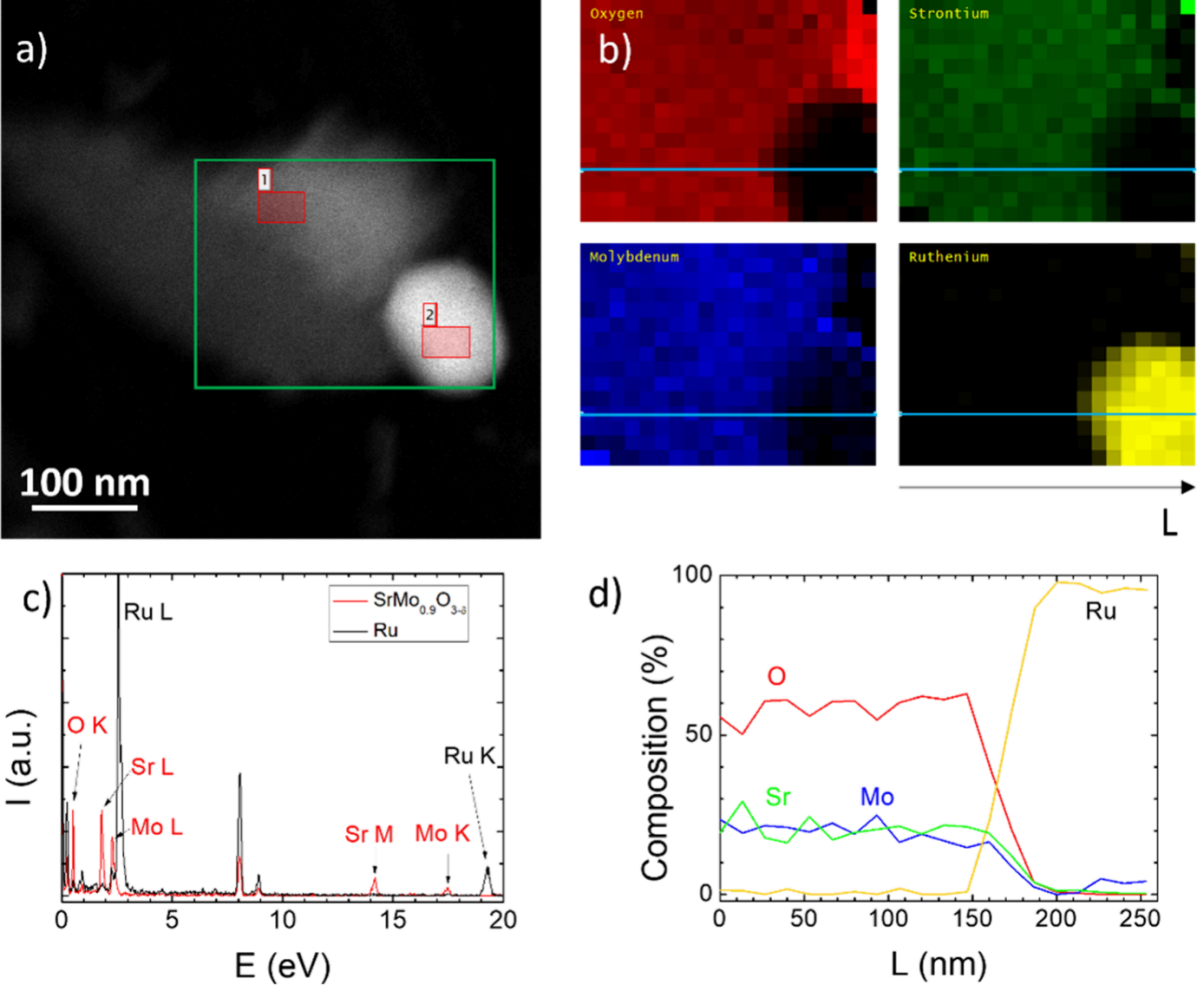
(a) ADF image of the crystallites shown in Figure 4a with the area (green rectangle) where EDS is taken. (b) Elemental maps based on the EDS spectra. (c) Two representative spectra for SrMo_0.9_O_3−δ_ (region 1 in (a)—red) and Ru (region 2 in (a)—black). (d) Elemental profiles along the cyan line in (b).

Figure 5a shows the same grains in the ADF image. As can be seen, the contrast in ADF imaging is just the opposite: The heavier elements are brighter, and the lighter elements are dimmer. The green rectangle depicts the area that is scanned for electron-dispersive X-ray spectroscopy. The two spectra shown in Figure 5c are taken from areas (1) and (2) in Figure 5a. The red spectrum belongs to pure SrMo_0.9_O_3−δ_; the black spectrum to (almost) pure Ru grain. The arrows with labels indicate elemental peaks of our material (the carbon and copper peaks coming from the grid are not considered). The elemental maps based on the oxygen K (0.52 keV), strontium L (1.81 keV), molybdenum L (2.29 keV), and ruthenium L (2.56 keV) peaks are shown in Figure 5b. These maps clearly show the separation of ruthenium from the SrMo_0.9_O_3−δ_ matrix. Figure 5d shows the profiles of each map along the direction depicted as the cyan line in Figure 5b. The ratio of elements in SrMo_0.9_O_3−δ_ is approximately Sr/Mo/O = 1/1/3, while only a small amount of molybdenum was observed in almost 100% ruthenium grain. The separation of ruthenium from the perovskite matrix can significantly enhance the performance of the test fuel cell, since Ru improves the catalytic activity.

### 3.3 Thermogravimetric Analysis (TGA)

**Figure 6:**
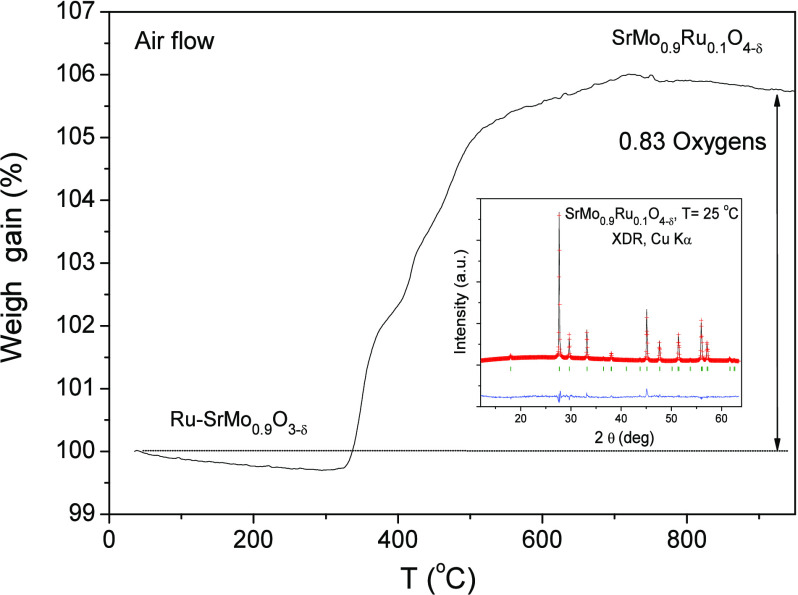
Thermal analysis curve for the Ru-SrMo_0.9_O_3−δ_ sample in air flow. Inset: Rietveld plot after the structural refinement from XRD data of the oxidation product yielding the SrMo_0.9_Ru_0.1_O_4−δ_ scheelite oxide. Observed (crosses), calculated (full line), and difference (at the bottom) XRD profiles in the tetragonal *I*4_*1*_*/a* space group. The vertical markers indicate the allowed Bragg reflections.

The evolution of the weight of the reduced Ru-SrMo_0.9_O_3−δ_ anode as the temperature increased from 25 to 900 °C in an air atmosphere was studied by TGA. The Ru-SrMo_0.9_O_3−δ_ sample started to gain weight around 325 °C. This fact means that the reduced perovskite with segregated Ru is oxidized to the scheelite phase with SrMo_0.9_Ru_0.1_O_4−δ_ composition (Figure 6). Ru atoms that appear segregated from the perovskite phase are introduced back into the scheelite structure as Ru^4+^ ions at the tetrahedral positions together with Mo atoms, when the sample is heated in air. This fact has been studied previously in other works as the reported by Nishihata et al*.*^21^ for Pd perovskites, or by Larralde et al.^17^ for Ni perovskites, where a similar phenomenon of Ni reincorporation into the scheelite oxide matrix is described when the reduced perovskite oxides are heated in air. As displayed in Figure 6, the weight gain corresponds to 0.83 O atoms/formula unit, thus obtaining an oxygen-defective scheelite structure of the SrMo_0.9_Ru_0.1_O_4−δ_ composition.

The inset in Figure 6 displays the XRD diagram obtained after TGA upon refinement of the defective scheelite crystal structure by the Rietveld method, showing the good agreement between the observed and calculated XRD patterns. The structure was refined in the *I*41*/a* space group (No. 88). Sr atoms are established in the center of the SrO_8_ polyhedra at 4*b* (0, 1/4, 5/8) sites, Mo and Ru atoms are randomly distributed at 4*a* (0, 1/4, 1/8) positions, and O oxygen atoms are situated at 16*f* (*x*, *y*, *z*) positions. The cell parameters obtained after the refinement were *a* = *b* = 5.3980(4) Å and *c* = 12.041(1) Å. Comparing these values with the parameters of the parent compound SrMoO_4_ (*a* = *b* = 5.3944 Å and *c* = 12.02 Å^22^), a subtle expansion of the structure is appreciated due to the bigger ionic radius of Ru^4+^ (0.62 Å) versus 0.59 Å of the Mo^6+^ ions in the same configuration,^23^ proving that the Ru atoms are introduced at the Mo position as Ru^4+^ ions as SrMo_0.9_Ru_0.1_O_4−δ_. A full reduction back to the Ru-SrMo_0.9_O_3−δ_ perovskite is obtained after heating again the sample in a 5% H_2_ atmosphere, demonstrating the full reversibility required in redox cycles, which is a very important characteristic that SOFC anode materials must have in SOFC.

### 3.4 Dilatometric Measurements

**Figure 7:**
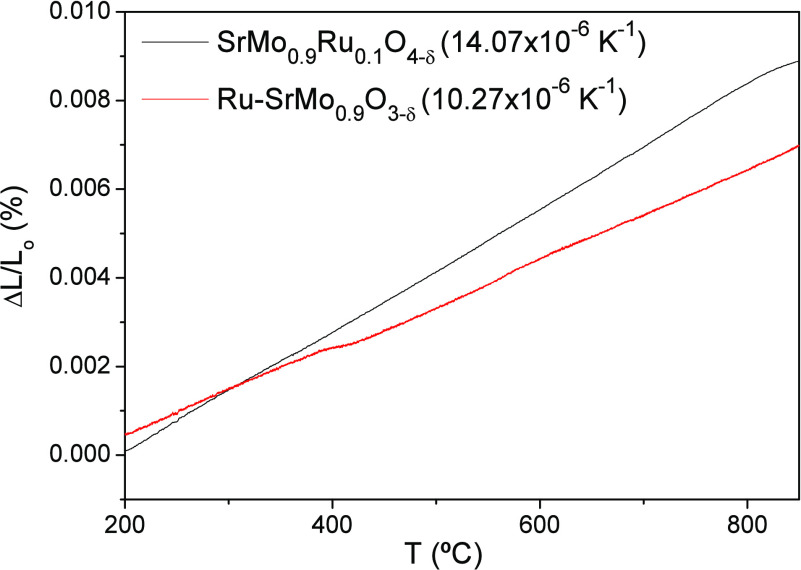
Thermal expansion determined by dilatometry of the SrMo_0.9_Ru_0.1_O_4−δ_ and Ru-SrMo_0.9_O_3−δ_ phases in air or in reducing the H_2_/N_2_ flow, respectively.

The main objective of this analysis is to determine the TECs of Ru-SrMo_0.9_O_3−δ_ perovskite in a 5% H_2_/95% N_2_ atmosphere and SrMo_0.9_Ru_0.1_O_4−δ_ scheelite in air, to compare them with the TEC bibliographic values of other components of the cell. Cracking problems in the test cell could occur during the heating and cooling operation cycles if the TECs of the reduced and oxidized samples are very different between them or with the electrolyte and LDC values. Figure 7 presents the dilatometric analysis of both materials from 200 to 900 °C. They both display a linear expansion, and no abrupt changes in the entire range of temperature were measured. TEC values of 14.07 × 10^–6^ K^–1^ and 10.27 × 10^–6^ K^–1^ for the perovskite and scheelite phases, respectively (Figure 7), are comparable to those presented by the LSGM electrolyte and the LDC buffer layer (12.50 × 10^–6^ K^–1^^24^ and 13.4 × 10^–6^ K^–1^,^25^ respectively). This means perfect mechanical compatibility between all the components of the cell as the temperature increases.

### 3.5 Electrical Conductivity Measurements and Chemical Compatibility

**Figure 8:**
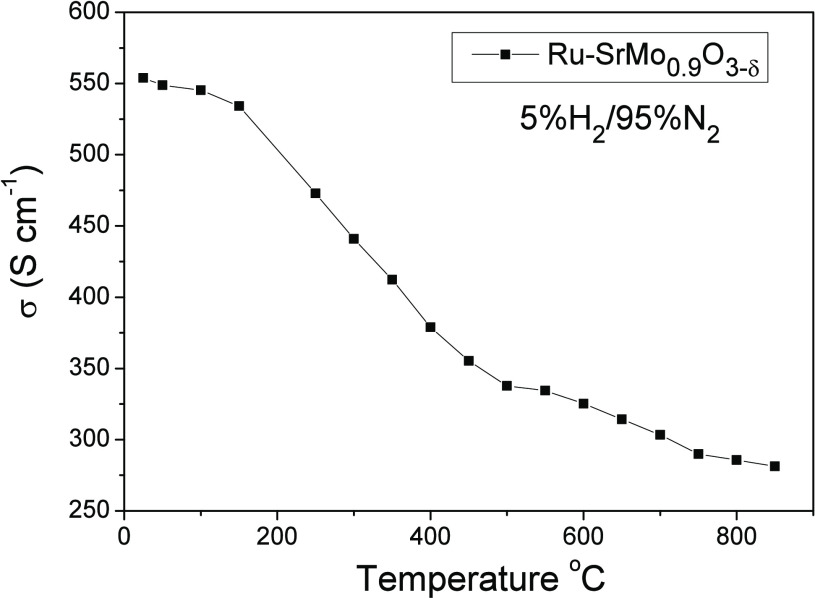
DC conductivity as a function of temperature for the Ru-SrMo_0.9_O_3−δ_ phase in reducing the H_2_/N_2_ flow.

Electrical conductivity measurements have been performed for Ru-SrMo_0.9_O_3−δ_ using the four-point method between 25 and 850 °C in a 5% H_2_/95% N_2_ atmosphere. The obtained result appears in Figure 8 showing a metallic behavior in the entire range of temperature measured. Maximum conductivities of 282 and 285 Scm^–1^ were obtained at 850 and 800 °C, working temperatures of an SOFC. These values are even better than other ones obtained for similar perovskites with Mo in the B position. For example, SrMo_0.9_Fe_0.1_O_3−δ_^12^ and SrMo_0.9_Cr_0.1_O_3_^13^ gave conductivities of 175 and 160 S cm^–1^, respectively, at 850 °C. A high electronic conductivity, above 100 S cm^–1^, is essential in an anode material, in order to deliver the electrical current to the external circuit.

**Figure 9:**
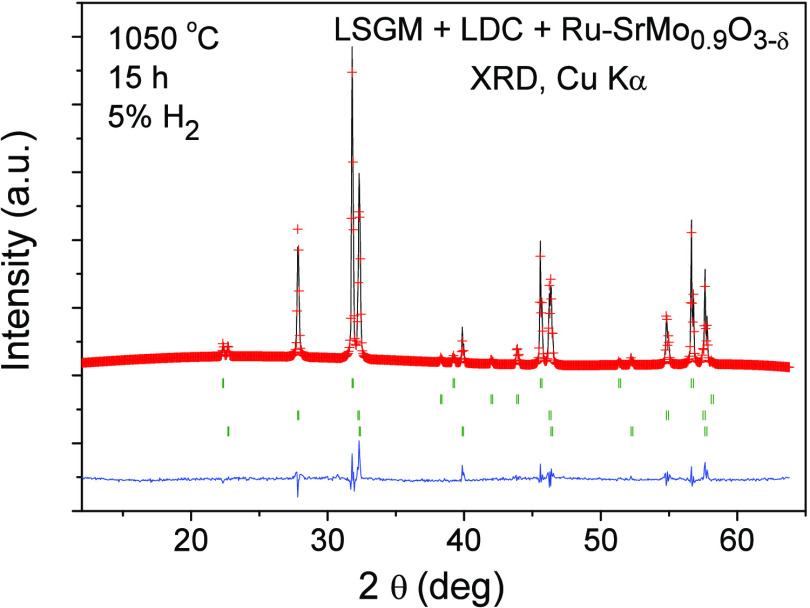
Rietveld-refined XRD profiles of a mixture of Ru-SrMo_0.9_O_3−δ_, LDC, and LSGM after a thermal treatment at 1050 °C in H_2_(5%)/N_2_, showing no reaction products between all phases other than the initial reactants. The four series of Bragg positions correspond to SrMo_0.9_O_3−δ_ perovskite, metallic Ru, LDC, and LSGM, respectively.

Another essential requisite to take into account in electrode materials to be used in an SOFC is the chemical compatibility of these with the other components of the cell. In this case, Ru-SrMo_0.9_O_3−δ_, LDC, and LSGM will be in close contact in the cell. For that reason, a mixture of finely ground Ru-SrMo_0.9_O_3−δ_, LDC, and LSGM was heated in a 5% H_2_ atmosphere at 1050 °C for 15 h and, after that, an XRD study was performed to probe those three phases that remained unaltered. Figure 9 shows an XRD diagram after the Rietveld refinement of the different crystal structures, showing no reaction products between all phases other than the initial reactants. The series of Bragg positions corresponds to SrMo_0.9_O_3−δ_ perovskite, metallic Ru, LDC, and LSGM, respectively. Therefore, we conclude that Ru-SrMo_0.9_O_3−δ_, LDC, and LSGM are chemically compatible at the working temperature of an SOFC.

### 3.6 Fuel Cell Performance

**Figure 10:**
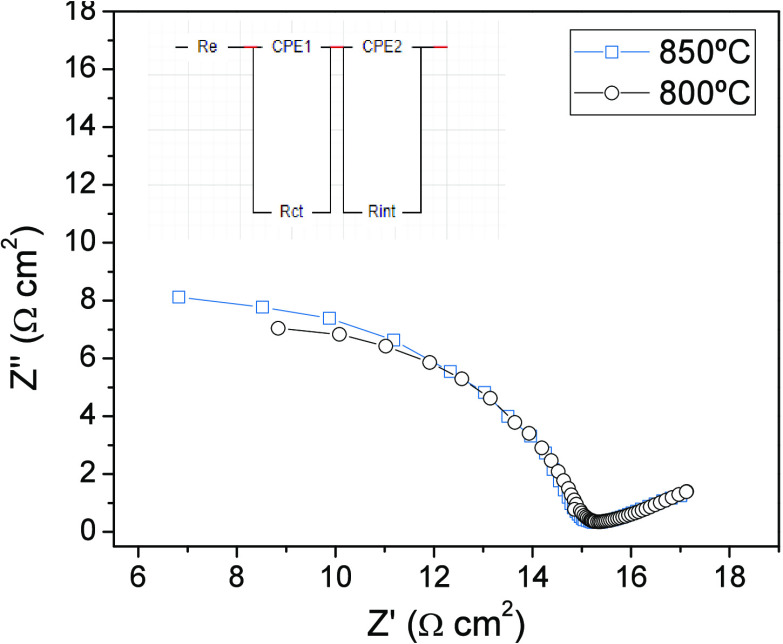
Electrochemical impedance spectra with the configuration Ru-SrMo_0.9_O_3−δ_/LDC/LSGM/SCFO measured in situ.

Electrochemical impedance spectroscopy (EIS) analysis of the single cell was conducted at both 800 and 850 °C, revealing a similar behavior at both temperatures (Figure 10). The obtained spectra were subjected to simulation employing a resistance and two series circuits. These circuits consist of a series of resistances (*R*_e_, *R*_ct_, and *R*_int_) and pseudocapacitors (CPE1 and CPE2), as depicted in the inset of Figure 10. *R*_e_ corresponds to the internal electrolyte resistance, with values of 15.2 and 15.0 Ω cm^2^ at 800 and 850 °C, respectively. *R*_ct_ represents the ionic and electronic charge transfer, with values of 0.5 and 0.6 Ω cm^2^ at 800 and 850 °C, respectively. These parameters are depicted in the first arc at high frequencies. *R*_int_, described within the second arc at low frequencies, simulates the resistance occurring at the electrode interface with the electrolyte, encompassing processes, such as anode hydrogen diffusion and cathode surface reactions.

**Figure 11:**
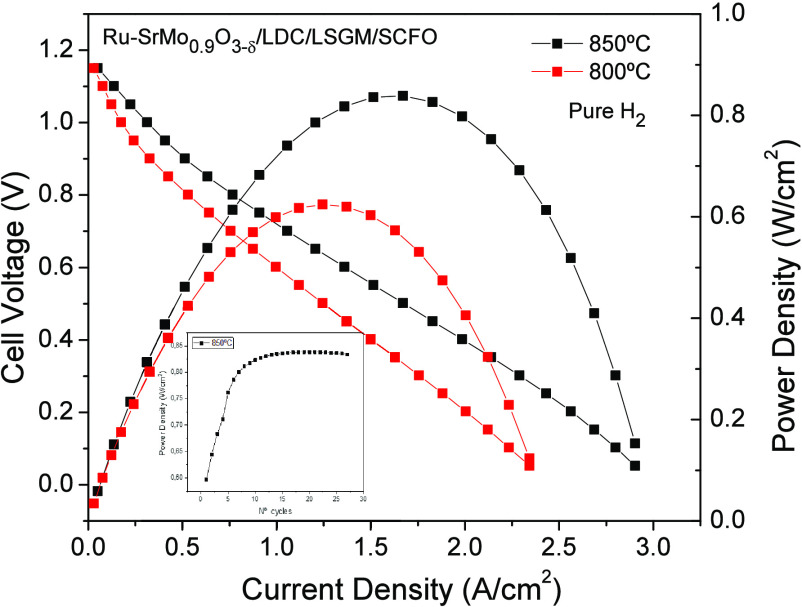
Cell voltage (left axis) and power density (right axis) as a function of the current density for the prepared single cell with the Ru-SrMo_0.9_O_3−δ_/LDC/LSGM/SCFO configuration in pure H_2_ flow at 850 and 800 °C. The inset shows the evolution of the power density as a function of the number of cycles at 850 °C.

A real SOFC single cell was built to check the performance of Ru-SrMo_0.9_O_3−δ_ as the anode. An electrolyte-supported configuration was chosen using a 300 μm-thick LSGM electrolyte pellet. LDC and SCFO were selected as the buffer layer and cathode, respectively. Figure 11 displays the power density curve of the Ru-SrMo_0.9_O_3−δ_ single cell operated with pure H_2_, displaying the cell voltage (*V*) (left axis) and power density (W/cm^2^) (right axis) as a function of current density at 800 and 850 °C. Figure 11 shows a high OCV of 1.15 V, meaning a good sealing of the cell and a dense electrolyte. The maximum power densities achieved by the cell were 625 and 840 mW/cm^2^, respectively.

The inset in Figure 11 depicts the variation of power density over the number of cycles at 850 °C. Initially, the output power rises steadily within the first 10 cycles while the complete reduction of the initially deposited oxidized scheelite phase in the anode material is achieved. This reduction process corresponds to the activation of the cell. Subsequently, after surpassing 25 cycles, the power density stabilizes consistently above 840 mW/cm^2^.

Table 2 gathers power density values obtained for other similar systems with the same composition such as SrMo_0.9_M_0.1_O_3−δ_ (M = Cr, Co, Fe, Mg, Ga) or Ni-Sr_0.9_Mo_0.9_O_3−δ_ at 850 °C using the same SCFO cathode material and the same LSGM electrolyte in the test cell, fed by H_2_. It can be observed that the power density obtained by Ru-SrMo_0.9_O_3−δ_ at the same temperature is very similar and even better in some cases, confirming an excellent performance of this material as an anode in SOFCs.

**Table 2:** Power Density Values for Other Similar Systems such as SrMo_0.9_M_0.1_O_3−δ_ (M = Cr, Co, Fe, Mg, Ga) or Ni-Sr_0.9_Mo_0.9_O_3−δ_ anodes at 850 °C

anode material	power density
SrMo_0.9_Cr_0.1_O_3−δ_	695 mW cm^–2^^13^
SrMo_0.9_Co_0.1_O_3−δ_	793 mW cm^–2^^15^
SrMo_0.9_Fe_0.1_O_3−δ_	874 mW cm^–2^^12^
SrMo_0.9_Mg_0.1_O_3−δ_	887 mW cm^–2^^16^
SrMo_0.9_Ga_0.1_O_3−δ_	907 mW cm^–2^^14^
Ni-Sr_0.9_Mo_0.9_O_3−δ_	1025 mW cm^–2^^17^
Ru-SrMo_0.9_O_3−δ_	840 mW cm^–2^[this work]

### 3.7 Postmortem Scanning Electron Microscopy

**Figure 12:**
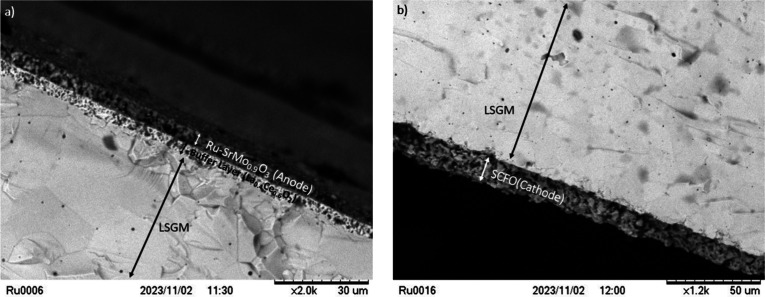
SEM micrograph showing (a) the porous anode layer of Ru-SrMo_0.9_O_3−δ_ and the buffer layer fully adhered to the dense electrolyte and (b) the SCFO cathode layer adhered to the LSGM electrolyte.

The SEM images shown in Figure 12 depict the cross-sectional view of the anode–electrolyte–cathode interface postmortem, after the cell operation. The arrangement of the anodic side is clearly displayed in Figure 12a: The porous anode layer, found to have a thickness of roughly 6–8 μm, is deposited on a buffer layer overlaid on top of the electrolyte LSGM layer. The essential porosity of the anode, allowing for hydrogen flow, is evident. The electrolyte, on the other hand, appears dense with no delaminations or fractures. In Figure 12b, the cathodic material appears to adhere to the electrolyte layer, much like the anodic side; the cathode layer possesses a porous structure with a thickness of approximately 16 μm. The basic arrangement necessary to accomplish their roles as electrode materials seems to remain unaffected even after undergoing single-cell tests at 850 °C.

## 4 Conclusions

A new material formed by a SrMo_0.9_O_3−δ_ perovskite matrix with segregated Ru-metal nanoparticles has been prepared and tested as an anode in SOFC at 800 and 850 °C, obtaining a maximum power density of 840 mW/cm^2^ with pure H_2_ as a fuel. This excellent performance as anode material is due to the MIEC properties, involving a high electronic conductivity of 282 Scm^–1^ at 850 °C combined with the sizable oxygen deficiency of the perovskite structure, which results in a substantial ionic conductivity across the solid. In addition, segregated Ru provides good catalytic activity for the HOR, which also enhances its properties as anode. NPD, SEM, and STEM techniques have helped to unveil the crystal structure and microstructure of the sample, which belong to a cubic deficient perovskite in B and O sites with the *Pm-*3*m* space group. SEM and STEM allow visualizing the segregated Ru-metal nanoparticles, as well as the absence of Ru within the perovskite oxide matrix. The TECs of 14.07 × 10^–6^ and 12.27 × 10^–6^ K^–1^ for the oxidized and reduced forms are perfectly compatible with the used buffer layer and electrolyte, avoiding cracking problems in the test cell during its operation as an energy conversion device. Finally, a postmortem SEM study confirmed that the Ru-SrMo_0.9_O_3−δ_ anode material does not appear to degrade after working at 850 °C during single-cell tests and it presents high porosity, which allows hydrogen to penetrate through the solid, enhancing the triple-phase boundary, where the HOR occurs. All these features are responsible for the substantial performance of this material as anode in IT-SOFCs.

## Supporting Information Available

SEM micrographs of the oxidized SrMo_0.9_Ru_0.1_O_4−δ_ sample showing the typical morphology; SEM micrographs of the reduced Ru-SrMo_0.9_O_3−δ_ sample; EDX results of the oxidized SrMo_0.9_Ru_0.1_O_4−δ_ sample; EDX results of the Ru particle; and EDX results of the perovskite matrix (PDF)
