# Supplementary material for: SrMo0.9O3−δ Perovskite with Segregated Ru Nanoparticles Performing as Anode in Solid Oxide Fuel Cells
Source: ACS Appl Mater Interfaces. 2024 Apr 2;16(14):17474–82. doi: 10.1021/acsami.3c19099 (PMC11009920; doi:10.1021/acsami.3c19099)
Supplement: Supplementary file 1 — am3c19099_si_001.pdf [file am3c19099_si_001.pdf]

SUPPLEMENTARY INFORMATION

$SrMo_{0.9}O_{3-\delta}$ perovskite with segregated Ru nanoparticles performing as anode in solid oxide fuel cells

Vanessa Cascos[1,2*], Mónica Chivite Lacaba[1,2], Neven Biskup[3], María Teresa Fernández-Díaz[4], José Antonio Alonso[2]

[1]Departamento de Química Inorgánica, Universidad Complutense de Madrid, E-28040, Madrid, Spain.
[2]Instituto de Ciencia de Materiales de Madrid, C.S.I.C., Cantoblanco, E-28049 Madrid, Spain.
[3]Departamento de Física de Materiales & Instituto Pluridisciplinar, Universidad Complutense de Madrid, 28040 Madrid, Spain
[4]Institut Laue Langevin, BP 156X, F-38042 Grenoble, France.
* Corresponding authors: vcascos@ucm.es

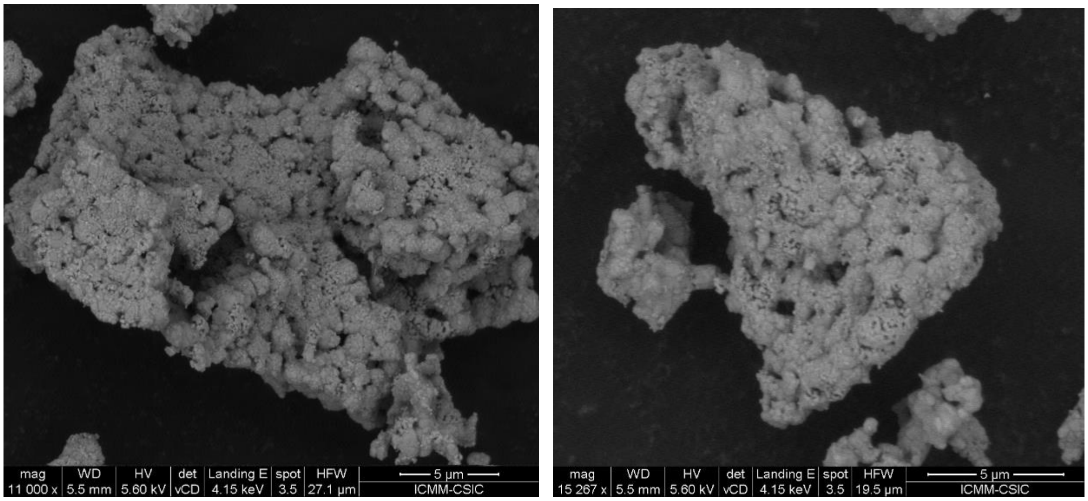

Fig. S1. SEM micrographs of the oxidized $SrMo_{0.9}Ru_{0.1}O_{4-\delta}$ sample showing the typical morphology, consisting of porous agglomerates of smaller particles with typical size of 1 µm, with different magnifications: (a) 11000 X, (b) 15267 X.

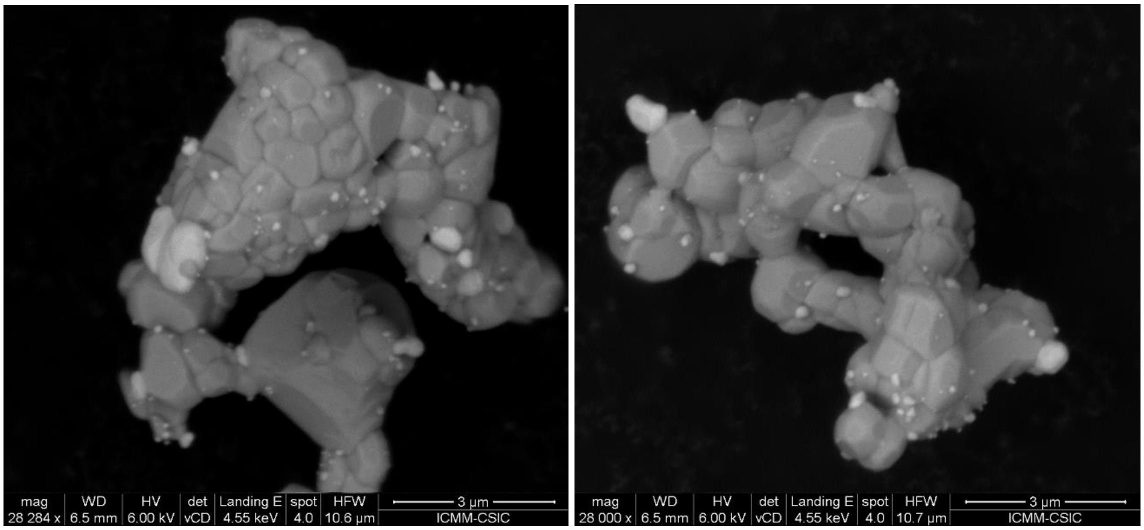

Fig. S2. SEM micrographs of the reduced Ru-$SrMo_{0.9}O_{3-\delta}$ sample, clearly showing the segregated Ru particles (white color), adhered to the surface of the perovskite oxide matrix (grey particles). Ru particles display a heterogeneous size, going from several nanometers to more than one micron. The EDX was performed in the large Ru particles shown in both images. Magnifications: (a) 28284 X, (b) 28000 X.

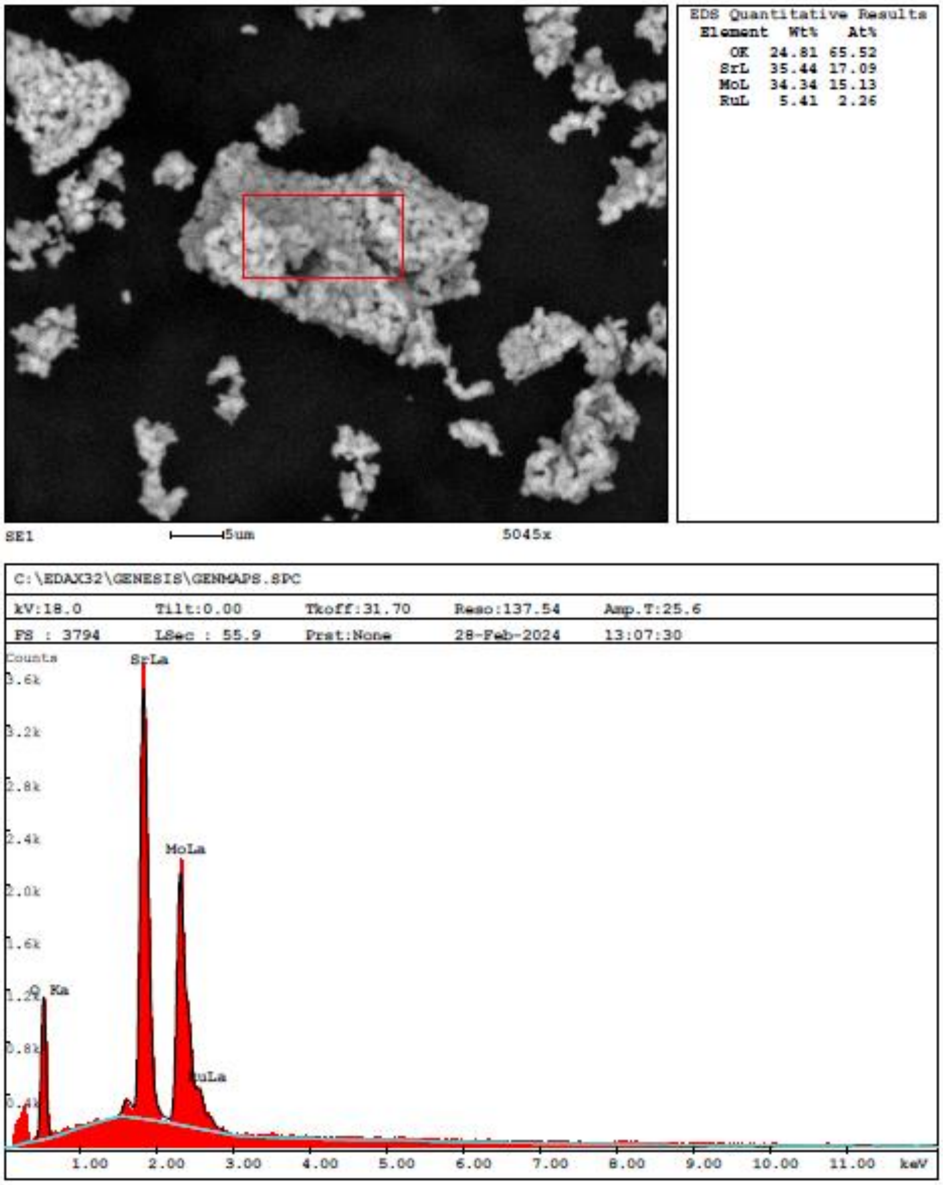

Fig. S3. EDX results of the oxidized $SrMo_{0.9}Ru_{0.1}O_{4-\delta}$ sample shown in the Figure (left top image). The ratio Sr:Mo:Ru is 1:0.87:0.13. This is consistent across different particles.

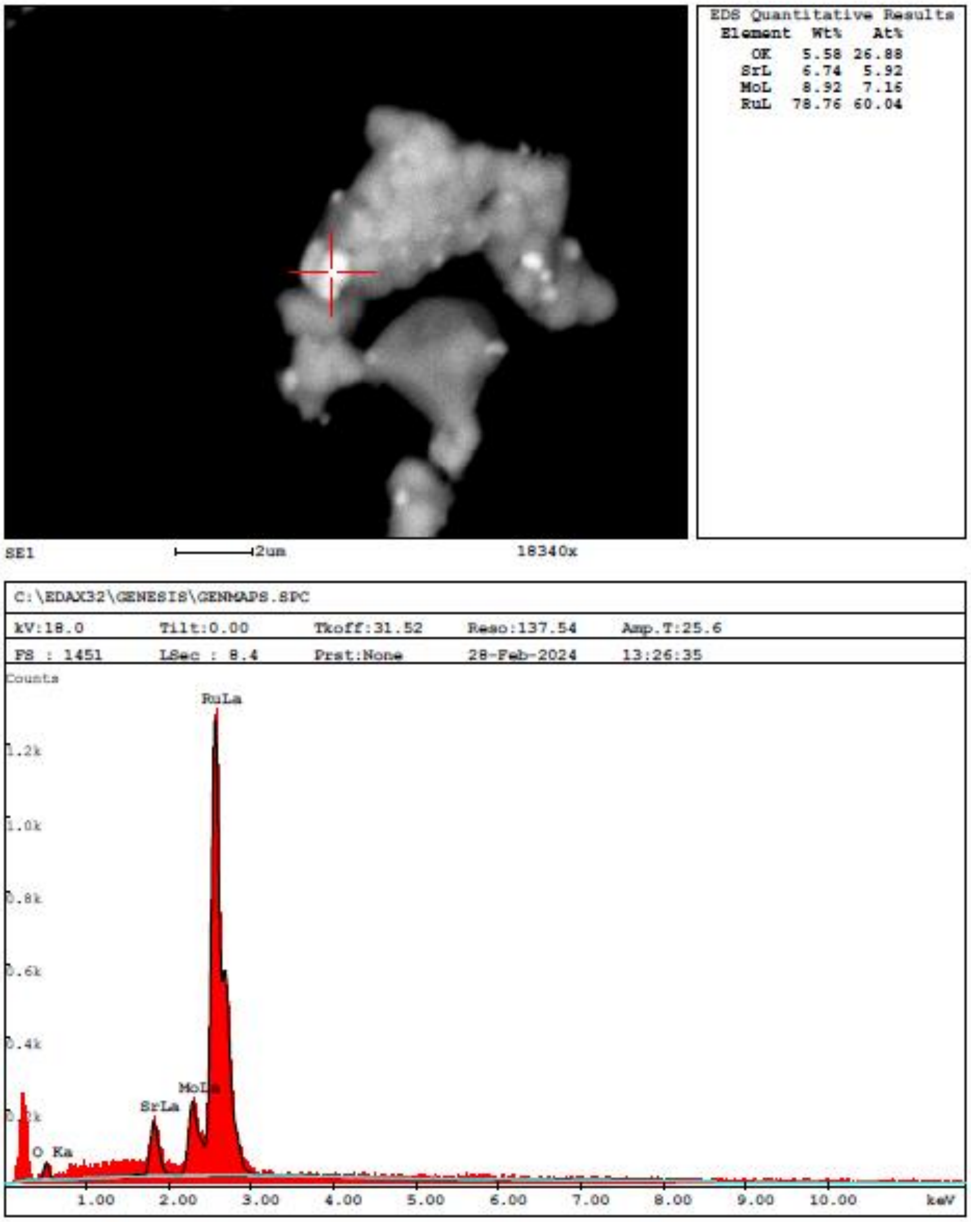

Fig. S4. EDX results of the Ru particle shown in the Figure (left top image). The spectrum shows a major Ru signal with a minor Sr, Mo and O peaks, due to the proximity of the perovskite oxide matrix and the finite width of the electron beam.

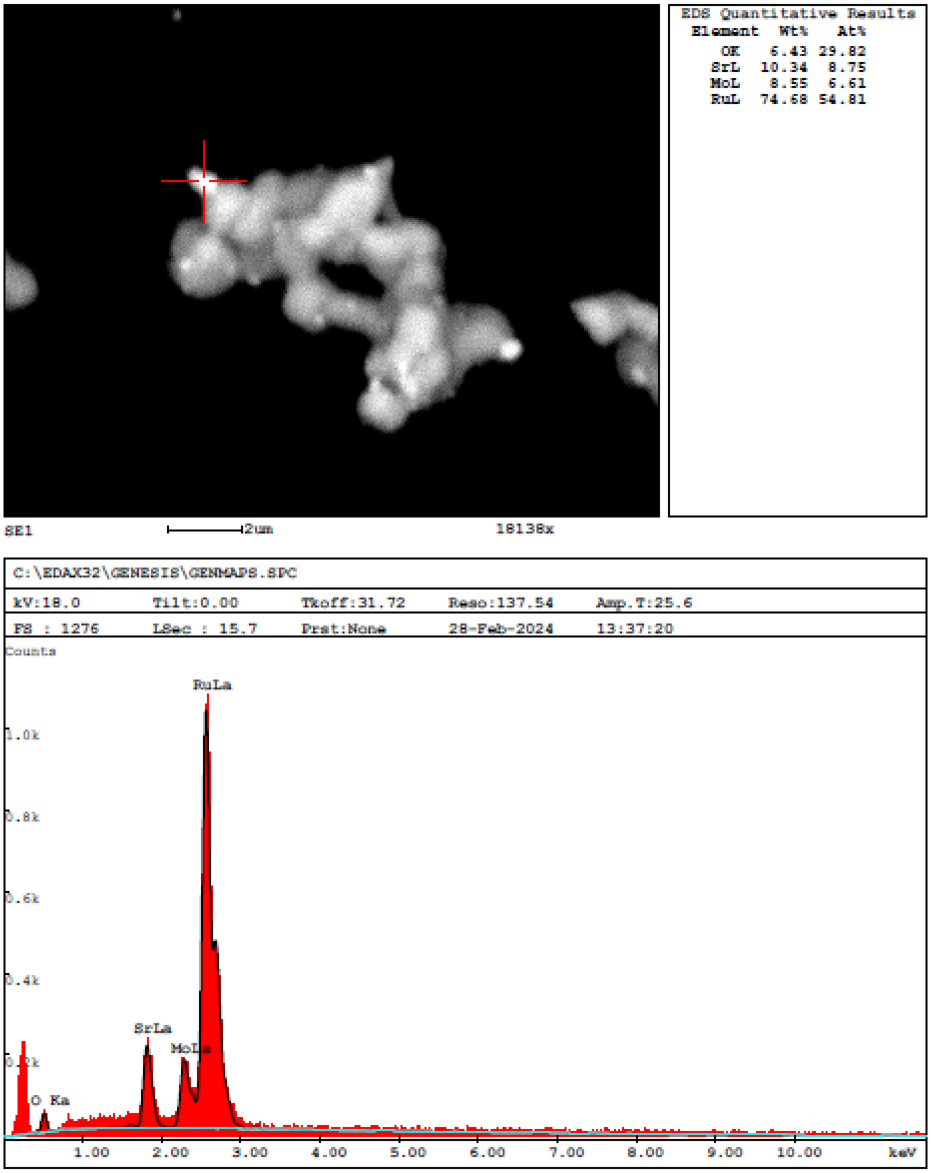

Fig. S5. EDX results of the Ru particle shown in the Figure (left top image). The spectrum shows a major Ru signal with a minor Sr, Mo and O peaks, due to the proximity of the perovskite oxide matrix and the finite width of the electron beam.

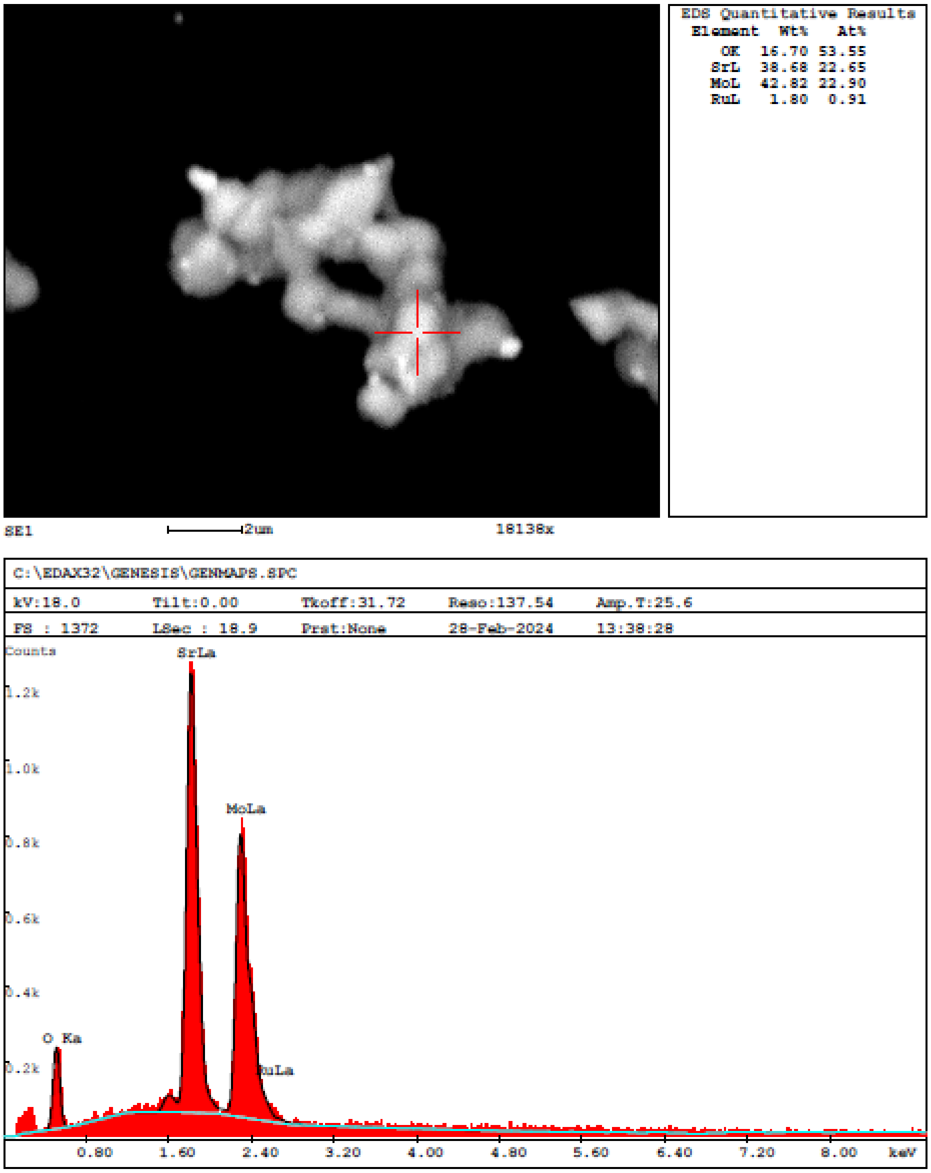

Fig. S6. EDX results of the perovskite matrix (left top image) in the reduced phase. The spectrum shows major Sr, Mo and O peaks, and a minor Ru signal due to the proximity of segregated Ru nanoparticles and the finite width of the electron beam.
